# The relationship between women’s experience of intimate partner violence and other socio-demographic factors, and under-5 children’s health in South Africa

**DOI:** 10.1371/journal.pone.0225412

**Published:** 2019-11-25

**Authors:** Sasha Frade, Nicole De Wet-Billings

**Affiliations:** Demography and Population Studies Programme, University of the Witwatersrand, Johannesburg, Gauteng, South Africa; Stellenbosch University, SOUTH AFRICA

## Abstract

Women in South Africa experience high levels of Intimate Partner Violence (IPV). There are numerous health consequences experienced by victims. However, children of IPV victims often experience negative emotional and developmental outcomes as well. In South Africa, infant and child health outcomes are not optimal and IPV is high, and thus there is a need to determine whether a relationship between them exists. This study used the 2016 South African Demographic and Health Survey. Mothers aged 15 to 49 and who were included in the Domestic Violence module formed the study population. Frequency tables and graphs were done, and unadjusted and adjusted logistic regressions were performed with each of the three reported child health outcomes (birth weight, duration of breastfeeding and diarrhoea incidence), IPV and other socio-demographic factors. Thirteen percent of women have experienced IPV. Five percent of their children were low birth weight, 10% had experienced diarrhoea; but 87% had been breastfed for 6+ months. Mothers in the rich wealth category were 37% more likely to have a child born at low birth weight but those aged 20 to 39 had around a 60% less likelihood of breastfeeding for 6+ months than 15 to 19-year olds. Women who had experienced IPV had around 77% higher odds of having a child experience diarrhoea in the last 2 weeks. Wealthier mothers often have unhealthier lifestyle practices and behaviours, due to more disposable income which could account for lower birth weight children. Mothers in tertiary education and starting their professional careers are normally around 20 to 39 years and should be provided supportive structures to be allowed to breastfeed their children. The long-term emotional and developmental consequences to children of IPV victims are known, but we now know that there are also very immediate consequences to the health of these children as well.

## Introduction

Women are vulnerable to violence including assaults, sexual violence and homicide at random (by unknown perpetrators), within their communities, families and especially from their intimate partners. South Africa has one of the highest rates of intimate partner or domestic violence in the world with 50% of all murders of women being by their intimate partners at a rate of 8.8 per 100,000 population [1]. Death is not the only consequence of intimate partner violence (IPV), with research showing that victims are also known to suffer from depression [2, 3], suicidal ideation [4], the development of chronic pain injuries [3], severe reproductive health outcomes [3], and are more susceptible to HIV infection [3, 5].

Furthermore, it is not only the direct victims, women, who suffer negatively. Research has shown that children who witness IPV often perform poorly at school and male children are known to become perpetrators of IPV as adults too [6, 7]. Also, children in households where there is IPV are often victims themselves and report various types of physical, sexual and emotional violence by the same perpetrators as their mothers [8, 9]. Research in other countries has found a relationship between IPV and child health and well-being outcomes, but none of these were done in South Africa [10–12].

Negative foetal and infant health outcomes include low foetal birthweight, foetal abnormalities, including the development of permanent disabilities and even infant and maternal death [13–15]. These outcomes are related to poverty and the inability to afford maternal nutrition care, lack of access to clinics due to transport and infrastructure difficulties and cultural or educational barriers to accurate knowledge of maternal and infant health and nutrition [16–20]. In South Africa, 14.4% of male and 15.2% of female infants are born weighing less than 2,500 grams [21]. Furthermore, infant mortality rates are currently at 36 per 1,000 live births and maternal mortality rates are 261 deaths per 100,000 live births [22, 23]. Related to these outcomes are cases of diarrhoea, and breastfeeding [24, 25]. Both are important health indicators for young children.

In a country like South Africa, where IPV is high and child health outcomes are poor, there is need to examine if an association between violence experienced by the mother and child health exists. This research will be needed for two reasons. First, we do not know the full extent of the consequences of IPV including those to children who may be indirectly affected. Second, South Africa and other countries in the region pledged to meet the Sustainable Development Goals, including the goal on improved child survival and maternal health, both targets South Africa failed to meet for the Millennium Development Goals [26]. In order to improve on past failures to meet goals, more research is needed on the factors that prevent optimal maternal and child health.

## Materials and methods

### Study design

This study used a secondary data source, namely the 2016 South African Demographic and Health Survey (SADHS). The SADHS is a nationally representative cross-sectional survey and follows from the DHS conducted in 1998.

In general, data from the DHS are comparable across countries, and is collected in over 85 developing countries, including those in sub-Saharan Africa. These surveys include many indicators on population, health and nutrition; and cover a broad array of other topics. Furthermore, the DHS includes a Domestic Violence module for several sub-Saharan African countries, including South Africa.

The 2016 SADHS took place between the 27^th^ of June and 4^th^ of November 2016 and covered all nine (9) of the South African provinces. A Master Sampling Frame (MSF) was used, created with the Enumeration Areas (EAs) of the 2011 South African Census. From the MSF Primary Sampling Units were selected, and from these Dwelling Units or Households were identified to be included in the survey. All women of reproductive age, as well as males (15–59 years) in the household, were included in the survey. Women were also asked questions regarding children born to them. For the Domestic Violence module, women were only included if consent was given and privacy could be obtained. If either consent and / or privacy could not be obtained, the woman was not included in the Domestic Violence module.

### Population and sample

A total of 8,514 women of reproductive age were included in the 2016 SADHS. However, only 4,357 women who had at least one child, were selected and interviewed as part of the Domestic Violence module.

To extract all required variables on child health outcomes, the individual (women’s) and child recodes were merged.

### Variables identification

#### Dependent variables

There are three outcomes that were used in order to assess pregnancy, post-partum and longer-term child health and well-being. The first outcome was whether the child was born at a low birth weight (0) or above 2.5 kgs (1). This outcome assesses the pregnancy-time period as the ultimate and measurable outcome of the child’s health and well-being in-utero.

The WHO recommends that mothers breastfeed their children exclusively for at least the first 6 months of life, and to try continuing breastfeeding thereafter for as long as possible. This recommendation has been made due to the numerous health benefits of breastfeeding for the child [27]. As such, duration of breastfeeding was included as the post-partum health and wellbeing indicator for the child. Those mothers who had not breastfed their last child were coded as (0), those who breastfed for 5 or less months were coded as (1) and those who breastfed for at least the minimum recommended time period of 6 months were coded as (2).

The final health and well-being indicator for the child was whether the child had experienced diarrhoea in the last two weeks. Those that had not experienced diarrhoea in the last 2 weeks were coded as (0), those who had experienced diarrhoea were coded as (1), and those mothers who did not know whether their last-born child had diarrhoea in the last 2 weeks were coded as (2). The variable was selected as the longer-term assessment of child health and well-being because diarrhoea is associated with both parasitic infections and nutritional deficiencies [28].

#### Independent variables

The independent variable of interest in this study was ever experience of IPV by the current partner (whether physical or sexual) of the mother of the reference child. Within the domestic violence module of the DHS questions were asked about physical and sexual abuse experienced; although physical IPV was separated into less severe and more severe physical IPV. All three are calculated using a dichotomous response (yes or no). Questions relating to experience of sexual IPV asked the respondent whether they had experienced unwanted sex, other unwanted sexual acts or physically coerced to perform sexual acts they did not want to by their partner. For more severe physical IPV, responses included whether a woman had ever been kicked / dragged, strangled / burned, or threatened with knife / gun / another weapon. Whilst for less severe physical IPV responded included whether a woman had ever been pushed, shaken, or had something thrown at them; slapped; punched; twisted / hair pulled by her partner. For the purposes of this study, if a woman had experienced less severe physical, more severe physical and/or sexual violence the response was taken as “having experienced IPV” (1). If the woman had never experienced either of these forms of IPV the response was coded as (0). A reliability test (Cronbach Alpha) was run on the sexual IPV, less severe IPV and more severe IPV yielding a scale reliability coefficient of 0.71.

Furthermore, the analysis included several other socio-demographic and “Child Wantedness” factors [Current Contraceptive Method Type and Wanted Last Child]. Child Wantedness factors were included in order to assess whether mother’s desire for children now (current contraceptive method—none, traditional or modern) and in the last pregnancy (wanted last child—wanted then, wanted later, wanted no more) could affect the health and well-being of the child. For contraceptive methods, traditional methods included periodic abstinence, withdrawal and country-specific traditional methods of proven effectiveness, and folk methods. On the other hand, modern methods include female sterilization, the contraceptive pill, IUDs, injectables, implants, female condom, male condom, diaphragm, contraceptive foam and contraceptive jelly, lactational amenorrhea method (LAM), standard days method (SDM), country-specific modern methods and if respondent-mentioned any other modern contraceptive methods.

Other selected socio-demographic variables included age of the mother (continuous variable), highest educational level (None, Primary, Secondary and Post-Secondary), Province, and Wealth (in quintiles). The variables were derived from past literature that was reviewed, having influenced child health and well-being as well as on women’s experience of IPV.

### Methods of data analysis

Frequency tables and graphs were used to show the distribution of women and children according to the dependent and independent factors, together with the Pearson Chi2 test of association. Furthermore, a table showing the percentage distributions and the Pearson Chi2 test for association between each of the original types of IPV (less severe physical, more severe physical and sexual) and each of the health outcomes has been shown. Thereafter unadjusted and adjusted logistic regression, with odds ratios, was used for all three outcomes: child having diarrhoea in the past two weeks, duration of breastfeeding of the last-born child, and birth weight of the last-born child. These analyses were done to ascertain the level of association between the selected independent variables (including experience of IPV) with each child health and well-being outcome.

Stepwise procedures (a combination of backward and forward selection) was used in order to assess which of the variables provided the best possible model to explain the association between IPV and children’s health outcomes. The final and accepted models, and selected variables, were included in an unadjusted and adjusted logistic regression for each of the health outcomes assessed. Furthermore, a Pearson Goodness of Fit Test was conducted on each of the models. Where the goodness of fit statistics was not significant, it was concluded that the data fit the model reasonably well. For all three adjusted models the p-value for the goodness-of-fit test was far higher than chosen significance level.

In relation to multi-collinearity, independent variables were checked for multicollinearity using Variance Inflation Factor (VIF) to detect whether any of the variables were correlated, if a variable had a VIF value of 10 or a tolerance level (1/VIF) of 0.1 or lower, then the variable was removed from the analysis. All VIF values were just over 1.01. Furthermore, a correlation matrix was computed to verify the possibility of multicollinearity, the highest value was 0.36 between province and place of residence (rural/urban), but insufficient to establish multicollinearity.

All analyses were done in Stata version 14.

### Ethical issues

The study analysed existing datasets using secondary data. Given that data collected was anonymised at the collation stage and no personal names or other identifiers were collected, respondents’ information remained anonymous and confidential. Therefore, ethical clearance was not required. The survey protocol for the 2016 SADHS itself, however, was reviewed and approved by the SAMRC Ethics Committee and the ICF Institutional Review Board.

However, intimate partner violence information is sensitive in nature. In order to ensure confidentiality and privacy, DHS data collectors were trained to only ask questions of women who provided consent and where privacy could be guaranteed. In the event that neither could be obtained, the survey did not continue. Furthermore, nurses accompanied the fieldwork team if assistance or support was required for any health-related components of the survey.

## Results

### Distribution of women of reproductive age by experience of IPV, socio-demographic factors and child-wantedness factors

**Fig 1:**
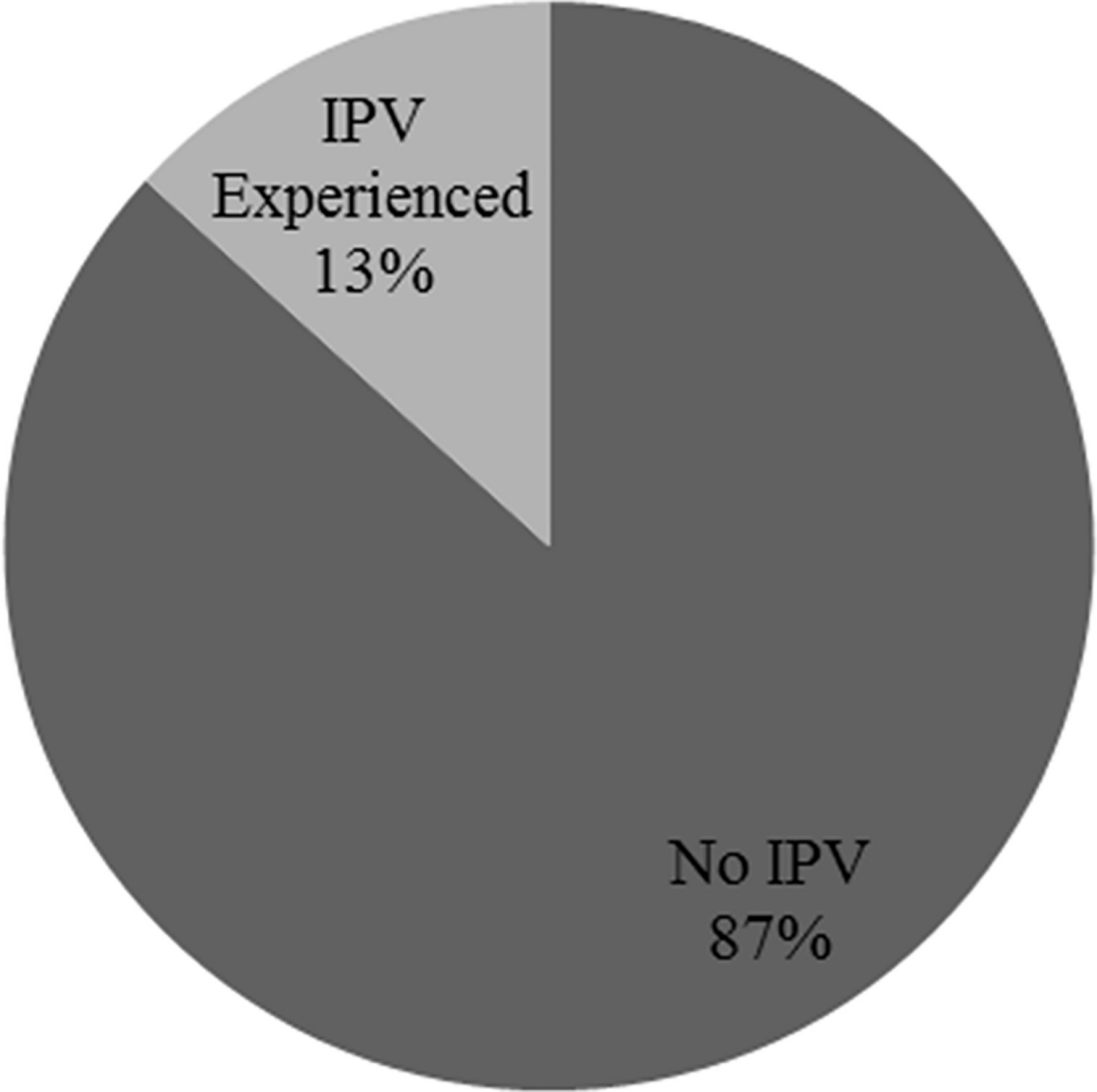
Experience of IPV of South African women of reproductive age.

Thirteen percent of South African women of reproductive age have experienced either or both physical and sexual intimate partner violence in their lifetime (Fig 1).

Table 1 shows the percentage distribution of the mother’s experience of the type of IPV (less severe physical, more severe physical and sexual IPV) by each of the child health outcomes. Pearson Chi2 tests showed significant association between less severe physical IPV and birth weight, as well as whether the child had diarrhoea in the last two weeks. Furthermore, more severe physical IPV was significantly associated with birth weight. Finally, sexual IPV showed a significant relationship with whether the child had diarrhoea in the last two weeks. Except in the case of sexual IPV and birth weight, for all other child health outcomes–mothers who has experienced each type of IPV showed higher percentages of negative health outcomes of the child compared to those who had not experiences these types of IPV.

**Table 1:** Percentage distributions of mother’s experience of type of IPV by child health outcome.

Type of IPV	Health Outcome							
	*Birth Weight*		*Breastfeeding Duration*			*Had Diarrhoea in the Last Two Weeks*		
	Low Birth Weight	Normal and Above	<1–5 months	6+ months	Never BF	No	Yes	Don't Know
*Less Severe Physical IPV*								
No	4.82	95.18	7.54	86.82	5.65	86.3	9.11	4.59
Yes	6.81	93.19	8.37	83.46	8.17	77.46	17.37	5.16
	Pearson chi2 = 3.701 p = 0.054		Pearson chi2 = 5.768 P = 0.056			Pearson chi2 = 14.076 P = 0.001		
*More Severe Physical IPV*								
No	4.83	95.17	7.64	86.58	5.78	85.61	9.68	4.7
Yes	7.72	92.28	7.72	84.27	8.01	79.71	15.94	4.35
	Pearson chi2 = 5.343 P = 0.021		Pearson chi2 = 2.761 P = 0.251			Pearson chi2 = 5.362 P = 0.068		
*Sexual IPV*								
No	5.18	94.82	7.61	86.38	6.01	85.78	9.66	4.56
Yes	2.11	97.89	8.45	86.62	4.93	64.71	27.45	7.84
	Pearson chi2 = 2.677 P = 0.102		Pearson chi2 = 0.393 P = 0.821			Pearson chi2(2) = 18.972 P = 0.000		

Table 2 shows the percentage and frequency distributions of South African women of reproductive age, by selected socio-demographic factors. Women were more or less equally distributed between the ages of 20 to 49, with a smaller percentage in the 15-19-year age category (5%). Most women (74%) had completed their secondary education, followed by those with a higher than secondary education (12%) and those who had only completed primary school (10%). Almost 60% of women of reproductive age, however, were living in urban areas around South Africa. Finally, almost half of all women were classified as poor at the time of the survey, whilst one-third were in the richer and richest wealth category.

**Table 2:** Percentage and frequency distributions of South African women of reproductive age by selected socio-demographic factors.

	Freq.	Percent
Age		
15–19	233	5.35
20–24	694	15.93
25–29	775	17.79
30–34	822	18.87
35–39	659	15.13
40–44	613	14.07
45–49	561	12.88
***Total***	***4357***	***100***
**Education Level**		
No Education	121	2.78
Primary Education	451	10.35
Secondary Education	3244	74.45
Higher Than Secondary	541	12.42
***Total***	***4357***	***100***
**Province**		
Western Cape	358	8.22
Eastern Cape	506	11.61
Northern Cape	383	8.79
Free State	426	9.78
KwaZulu-Natal	618	14.18
North West	433	9.94
Gauteng	478	10.97
Mpumalanga	541	12.42
Limpopo	614	14.09
***Total***	***4357***	***100***
**Place of Residence**		
Urban	2527	58.00
Rural	1830	42.00
***Total***	***4357***	***100***
**Wealth Category**		
Poorest	869	19.94
Poorer	1020	23.41
Middle	1025	23.53
Richer	861	19.76
Richest	582	13.36
***Total***	***4357***	***100***

Table 3 below shows that almost half of all women of reproductive age were on no form of contraception at all (48%), whilst just over half were on some form of modern contraception (51%). Less than 1% of women stated they were currently using traditional forms of contraception. Together, over half of all women of reproductive age stated that they either wanted their last-born child later (31%) or did not want the child at all (22%).

**Table 3:** Percentage and frequency distributions of South African women of reproductive age by “child wantedness” factors.

Contraceptive Method Type	Freq.	Percent
No method	2100	48.20
Traditional method	14	0.32
Modern method	2243	51.48
***Total***	***4357***	***100***
**Last Child Wanted**	** **	** **
Wanted then	784	46.50
Wanted later	530	31.44
Wanted no more	372	22.06
***Total***	***1686***	***100***

### Child health and well-being at birth: Birth weight

**Fig 2:**
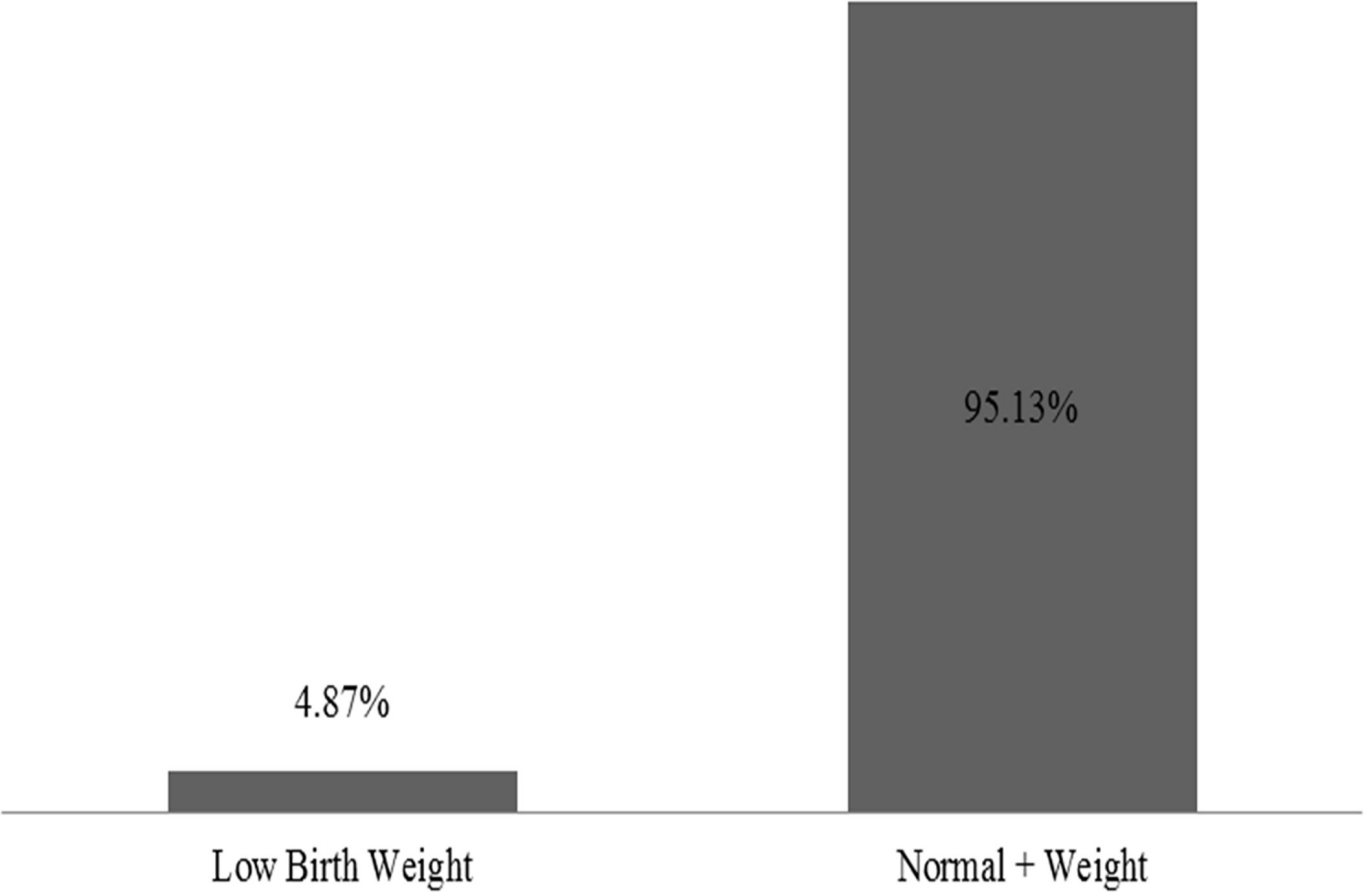
Percentage of South African U-5 children born at low and normal (and above) birth weights.

Fig 2 shows that around 5% of South African children under the age of 5 were low birth weight (less than 2.5 kgs) at birth.

Mothers who had experienced IPV had a 31% lower likelihood of having a child born at low birth weight, although this was not significant in the adjusted model (Table 4). Also, in the unadjusted model, as women’s age increased there was an increase in the likelihood of having a low birth weight baby. Those in the richest wealth category were 67% more likely to have a child born at low birth weight than those in the poorest wealth category in the unadjusted model only. On the other hand, those on traditional and modern forms of contraception were 86% and 36% less likely to have children born at low birth weights than those women on no form of contraceptive method in the unadjusted model, respectively. In the adjusted model, women on traditional forms of contraception were 81% less likely. However, the results of women on traditional contraception should be assessed with caution due to the extremely low number of women reporting to use this method type (<1%).

**Table 4:** Unadjusted and adjusted regression for low birth weight of last-born child.

	Unadjusted			Adjusted		
	OR	95% CI	P-Value	OR	95% CI	P-Value
**IPV**						
No	**RC**			**RC**		
IPV Experienced	0.69*	0.48–0.99	0.042	0.71	0.48–1.04	0.072
**Age**						
Age	1.05*	1.03–1.07	0.000	1.04	0.98–1.03	0.727
**Edu**						
No Edu	**RC**			**RC**		
Primary	0.43	0.13–1.46	0.177	0.72	0.20–2.66	0.626
Secondary	0.50	0.16–1.59	0.240	0.89	0.26–3.11	0.857
Higher	0.49	0.14–1.62	0.240	0.73	0.19–2.75	0.641
**Province**						
Western Cape	**RC**			**RC**		
Eastern Cape	1.28	0.65–2.49	0.472	1.87	0.88–3.96	0.103
Northern Cape	0.78	0.41–1.49	0.450	1.03	0.51–2.09	0.932
Free State	0.80	0.42–1.51	0.489	1.05	0.52–2.09	0.899
KwaZulu-Natal	0.76	0.42–1.37	0.362	0.90	0.46–1.75	0.756
North West	1.39	0.68–2.82	0.363	2.04	0.93–4.48	0.074
Gauteng	1.03	0.54–1.98	0.921	1.53	0.76–3.09	0.238
Mpumalanga	1.12	0.59–2.13	0.724	1.54	0.76–3.14	0.234
Limpopo	0.97	0.53–1.79	0.923	1.23	0.60–2.55	0.568
**Place of Res**						
Urban	**RC**			**RC**		
Rural	1.02	0.77–1.35	0.882	1.18	0.80–1.73	0.404
**Wealth Category**						
Poorest	**RC**			**RC**		
Poorer	1.05	0.73–1.53	0.794	1.03	0.67–1.59	0.884
Middle	1.42	0.46–1.05	0.096	1.28	0.80–2.06	0.304
Richer	1.27	0.41–1.02	0.256	0.96	0.57–1.61	0.879
Richest	1.67*	0.46–1.28	0.054	1.26	0.65–2.44	0.490
**Current Contraceptive Type**						
None	**RC**			**RC**		
Traditional	0.14*	0.04–0.52	0.003	0.19*	0.04–0.92	0.039
Modern	0.64*	0.48–0.85	0.002	1.14	0.83–1.56	0.421
**Wanted Last Child**						
Wanted	**RC**			**RC**		
Wanted later	0.88	0.63–1.23	0.468	0.94	0.65–1.36	0.74
Did not want	0.89	0.61–1.28	0.526	0.91	0.61–1.34	0.63

* Significant at the 95% confidence interval

### Post-partum health and well-being at birth: Duration of breastfeeding

**Fig 3:**
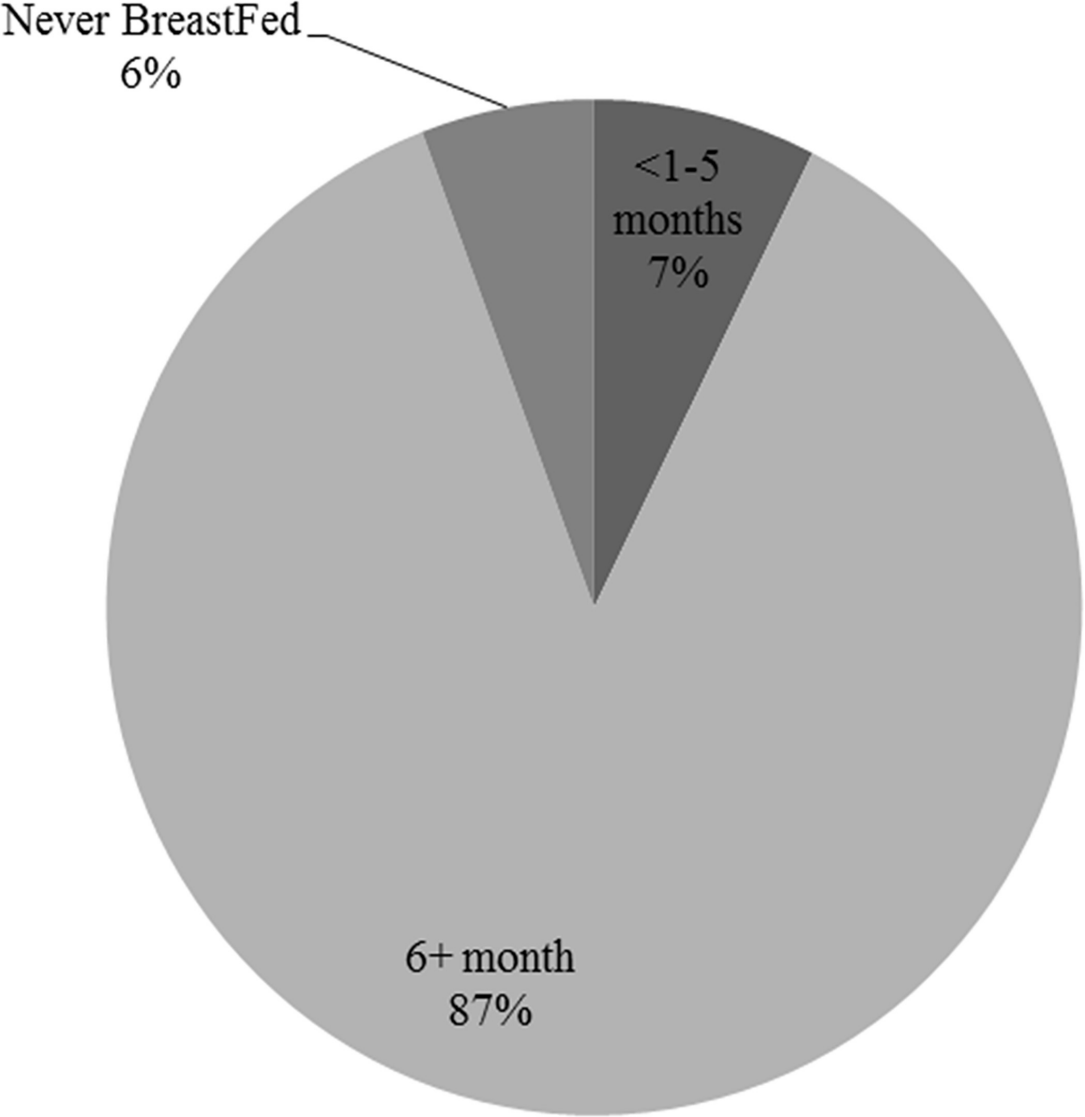
Duration of breastfeeding of last-born child amongst South Africa women of reproductive age.

Six percent of these children were never breastfed, whilst 7% were breastfed for less than the recommended 6 months. The remaining 87%, however, were breastfed for at least 6 months (Fig 3).

IPV experience of the mother did not show significant results with the duration of breastfeeding (Table 5). However, in the unadjusted model, as age of the mother increased the likelihood of breastfeeding for 6 months or longer increased significantly. These results, however, did not remain significant in the adjusted model.

**Table 5:** Unadjusted and adjusted regression for duration of breastfeeding of last-born child.

	Unadjusted			Adjusted		
	OR	95% CI	P-Value	OR	95% CI	P-Value
**IPV**						
No	**RC**			**RC**		
IPV Experienced	0.95	0.69–1.31	0.750	0.95	0.67–1.36	0.788
**Age**						
Age	1.04*	1.02–1.05	0.000	0.99	0.97–1.01	0.330
**Edu**						
No Edu	**RC**			**RC**		
Primary	0.76	0.31–1.87	0.550	1.18	0.43–3.25	0.756
Secondary	0.65	0.28–1.49	0.305	1.24	0.48–3.21	0.658
Higher	0.58	0.24–1.38	0.216	1.20	0.43–3.32	0.730
**Province**						
Western Cape	**RC**			**RC**		
Eastern Cape	1.17	0.71–1.92	0.540	1.11	0.61–2.01	0.733
Northern Cape	1.48	0.85–2.60	0.167	1.71	0.91–3.23	0.097
Free State	1.09	0.68–1.82	0.732	1.12	0.62–2.03	0.697
KwaZulu-Natal	1.05	0.66–1.68	0.825	1.06	0.60–1.88	0.843
North West	1.01	0.62–1.67	0.955	1.14	0.63–2.08	0.657
Gauteng	1.28	0.77–2.13	0.346	1.58	0.88–2.84	0.127
Mpumalanga	0.95	0.59–1.52	0.837	1.15	0.65–2.04	0.639
Limpopo	2.14*	1.25–3.67	0.006	2.33*	1.21–4.47	0.011
**Place of Res**						
Urban	**RC**			**RC**		
Rural	1.09	0.87–1.37	0.464	0.96	0.69–1.32	0.786
**Wealth Category**						
Poorest	**RC**			**RC**		
Poorer	0.84	0.58–1.21	0.348	0.75	0.50–1.13	0.172
Middle	0.72	0.50–1.03	0.076	0.55*	0.36–0.84	0.005
Richer	0.83	0.57–1.22	0.355	0.56*	0.35–0.90	0.016
Richest	0.62*	0.42–0.92	0.019	0.33*	0.19–0.56	0.000
**Current Contraceptive Type**						
None	**RC**			**RC**		
Traditional	0.33	0.07–1.50	0.152	0.77	0.14–4.18	0.763
Modern	0.54*	0.42–0.68	0.000	1.02	0.77–1.33	0.909
**Wanted Last Child**						
Wanted	**RC**			**RC**		
Wanted later	1.20	0.90–1.59	0.217	1.15	0.84–1.56	0.390
Did not want	1.10	0.80–1.50	0.560	1.15	0.82–1.61	0.421

* Significant at the 95% confidence interval

In the unadjusted and adjusted model women living in Limpopo province were over two times more likely to breastfeed their child for longer periods than women living in the Western Cape. Furthermore, in the adjusted model, women in the middle, richer and richest wealth categories were 45%, 44% and 67% less likely to breastfeed their children for longer periods than women who were poorest, respectively. Finally, women on modern contraception were 46% less likely to breastfeed their last born for longer periods than those who were on no contraceptive method in the unadjusted model.

### Longer-term child health and well-being at birth: Child experience of diarrhoea in the past 2 weeks

**Fig 4:**
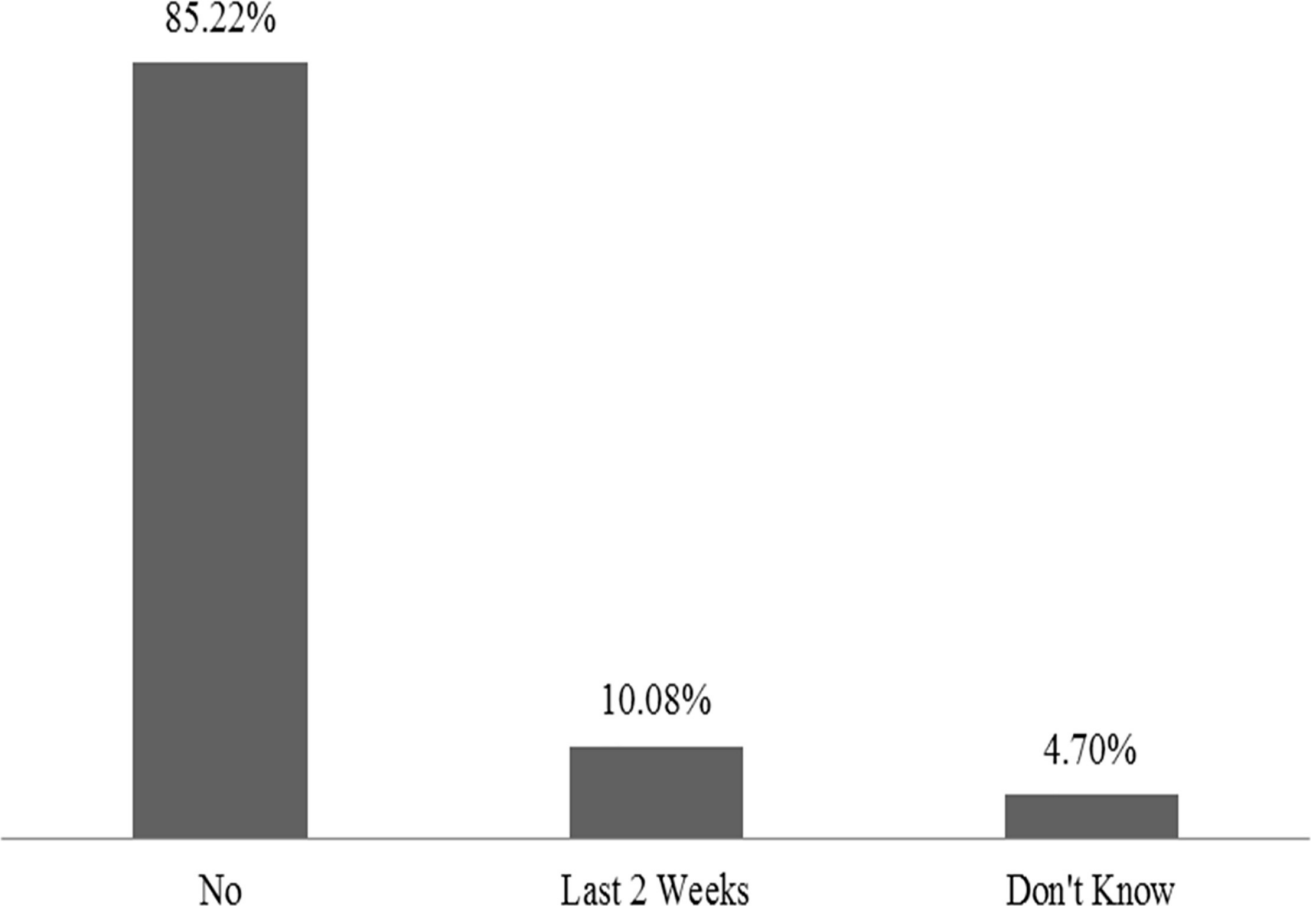
Percentage of under-5 children who had diarrhoea in the last two weeks.

Just over 10% of South African children under the age of five years had experienced diarrhoea in the past two weeks (Fig 4).

The table below (Table 6) shows the unadjusted and adjusted models for children who had diarrhoea in the last two weeks (current health and well-being). In both the unadjusted and adjusted models, women who had experienced IPV had 77% and 79% higher odds, respectively, than women who had not experienced IPV of having a child who had diarrhoea in the last 2 weeks. Furthermore, women living in Gauteng had over 2 times more likelihood of having children who had experienced diarrhoea in the past two weeks than women living in the Western Cape. Both in the unadjusted an adjusted model, as the age of the women increased the likelihood of having had a child with diarrhoea in the last two weeks decreased.

**Table 6:** Unadjusted and adjusted regression for diarrhoea in the last 2 weeks.

	Unadjusted			Adjusted		
	OR	95% CI	P-Value	OR	95% CI	P-Value
**IPV**						
No	**RC**			**RC**		
IPV Experienced	1.77*	1.25–2.50	0.001	1.79*	1.25–2.56	0.002
**Age**						
Age	0.97*	0.95–0.99	0.005	0.97*	0.95–1.00	0.022
**Edu**						
No Edu	**RC**			**RC**		
Primary	1.70	0.55–5.24	0.358	1.80	0.57–5.75	0.318
Secondary	0.92	0.31–2.69	0.875	0.89	0.29–2.73	0.842
Higher	0.65	0.20–2.09	0.475	0.63	0.18–2.15	0.459
**Province**						
Western Cape	**RC**			**RC**		
Eastern Cape	1.02	0.49–2.12	0.950	0.92	0.41–2.07	0.843
Northern Cape	1.01	0.47–2.18	0.982	0.97	0.42–2.24	0.952
Free State	1.01	0.47–2.16	0.974	1.13	0.50–2.56	0.765
KwaZulu-Natal	1.26	0.63–2.50	0.516	1.20	0.56–2.58	0.645
North West	1.41	0.70–2.87	0.339	1.36	0.62–2.99	0.442
Gauteng	2.11*	1.07–4.15	0.031	2.31*	1.11–4.81	0.025
Mpumalanga	1.46	0.74–2.88	0.276	1.38	0.64–2.94	0.409
Limpopo	1.33	0.68–2.62	0.403	1.35	0.62–2.96	0.454
**Place of Res**						
Urban	**RC**			**RC**		
Rural	1.09	0.83–1.44	0.519	1.06	0.74–1.54	0.738
**Wealth Category**						
Poorest	**RC**			**RC**		
Poorer	1.06	0.73–1.53	0.758	1.11	0.75–1.64	0.603
Middle	0.70	0.46–1.05	0.081	0.75	0.48–1.17	0.209
Richer	0.64	0.41–1.02	0.058	0.76	0.45–1.29	0.308
Richest	0.76	0.46–1.28	0.308	1.04	0.55–1.97	0.894
**Current Contraceptive Type**						
None	**RC**			**RC**		
Traditional	-	-	-	-	-	-
Modern	1.30	0.97–1.76	0.084	1.34	0.97–1.84	0.074
**Wanted Last Child**						
Wanted	**RC**			**RC**		
Wanted later	1.19	0.87–1.62	0.269	1.10	0.78–1.55	0.597
Did not want	0.97	0.67–1.39	0.864	1.00	0.68–1.47	0.984

* Significant at the 95% confidence interval

## Discussion

Over one in 10 South African women of reproductive age have experienced either physical or sexual IPV, or both, in South Africa. This is in line with reported levels of domestic abuse in South Africa, reported elsewhere [29]. On the other hand, there are studies conducted in South Africa that have reported much higher rates of intimate partner violence–these studies were not self-reported, but clinical samples or where there were visits to a medical doctor [30]. The difference could be explained by the sensitivity in reporting such incidents. South Africa has a notoriously low reporting rate of violence and violent acts [31] and respondents, although having provided consent and been in a private environment, may not have felt comfortable to report such acts to the fieldworker. Furthermore, studies have found that in the South African context women often accept such violence due to a patriarchal system, alcohol abuse, infidelity and failure to support children financially [32].

Subsequently, the direct and negative consequences of such abuse are well-known [2–5]. However, what has not been assessed until now is the negative health consequences that the experience of IPV can have on the child of the mother experiencing the abuse. Especially given the high violence and crime rates in the country.

The finding that women who are on contraception have a lower likelihood of having low birth weight babies speaks to the idea of child wantedness. Women who have decided to be on a contraceptive method have taken the decision to control the number of children they have. As such, once pregnant, women who have made the decision to become pregnant have the time and resources to ensure their health, well-being and optimal nutrition during pregnancy [33].

It is a positive result to see that almost 90% of South African women of reproductive age have breastfed their children for 6 months or longer. Breastmilk has been found to provide the most high-quality nutrients to the child, increasing child’s health and survival probabilities [27]. This result is far higher than those mothers reported to exclusively breastfeed in South Africa (at around 7%) [34] given that past the 6-month mark most mothers would have begun the weaning process. This study did not find a significant relationship between experience of IPV and breastfeeding, which is the opposite of what was found in the United States [35]. As such, research is needed to identify mothers who have exclusively breastfed and whether IPV influences their probability of completing the 6-month exclusive breastfeeding recommendation from the WHO.

The results found in the unadjusted model, that women who are aged between 20 to 39 years of age show a lower likelihood of breastfeeding for longer periods is not contrary to what we have seen in literature. Women in this age group are often either studying full-time at tertiary institutions or are just starting their professional careers. As such, many are not often with their child and are therefore not able to breastfeed [36]. Furthermore, in South Africa, many children live with extended family members so that mothers and parents are able to continue with studies and /or find better employment in other provinces [37, 38]. This could also explain the fact that those on modern contraceptives were found to have a lower likelihood of breastfeeding their children for longer periods. In these situations, mothers would not be able to breastfeed their children. Although the South African Department of Health actively encourages breastfeeding, more needs to be done to ensure that mothers situations facilitate children being with them for at least long enough that they can breastfeed them until the 6-month mark (and preferably longer), given the numerous health benefits associated with breastfeeding.

Diarrhoea is a childhood illness that is associated with numerous nutritional deficiencies and structural inequalities [28]. Given that the time-period in question was two weeks from the time of the survey, the fact that 10% of children under the age of 5 had experienced diarrhoea requires closer investigation in order to assess the reasons why this occurs. One of the reasons found in this study is IPV experienced by the mother. In these situations, the mother may not have had the state (due to pain or physical signs of abuse), money or transport (if such resources are being withheld), or the lack of autonomy to make the decision to take the child to the doctor. All of which has been found to be factors associated with women who experience abuse, in other studies [39]. The fact that wealthier women were found to have a lower likelihood of having children who experienced diarrhoea in the past two weeks corroborates this–as women with more resources can make these decisions regardless of the partner’s involvement easier than those who are dependent on the money or resources of the partner.

It is imperative that programmes and policies move towards decreasing the levels of violence and abuse experienced by women. The negative effects of such abuse have dire consequences for the women, but also for her children. The long-term emotional and developmental consequences to South African children from homes where abuse occurs are known [40], but we now know that there are also very immediate consequences to the health and well-being of these children as well. This study shows that it is crucial that the public health system incorporate programmes addressing intimate partner violence, even into the post-natal care and baby clinics, given that the health effects are felt by both women and their children. However, what this study was not able to show, and which could be extremely important, is to assess the experiences of such women and how the physical and mental implications of experience of violence affects child health. As such, qualitative studies about these experiences and quantitative studies providing indicators on the severity and frequency of violence, and the pathways in which this occurs, are required. Finally, studies of this nature are required in other settings in order to assess whether such experiences are seen throughout various contexts, or whether they are specific to South Africa.

### Limitations

This study has been important in showing that there is a relationship between women’s experience of intimate partner violence and immediate child health outcomes. However, several limitations are identified. First is that given the cross-sectional nature of the data, temporality could not be assessed. It is therefore not known whether the incident of violence, or multiple acts, were directly related to the negative child health outcome. Furthermore, the self-reported nature of the violent acts presents a bias which may have contributed to the lower levels of intimate partner violence than what has been previously reported in South Africa. Finally, intimate partner violence is a sensitive topic and although fieldworkers were extensively trained on how to ask such questions, respondents may not have felt comfortable to report on acts of violence perpetrated by their current partner.
